# Triple-negative and triple-positive breast cancer cells reciprocally control their growth and migration via the S100A4 pathway

**DOI:** 10.1080/19336918.2022.2072554

**Published:** 2022-05-12

**Authors:** Elena A. Dukhanina, Tatiana N. Portseva, Alexander S. Dukhanin, Sofia G. Georgieva

**Affiliations:** a Department of Transcription Factors, Engelhardt Institute of Molecular Biology, Russian Academy of Sciences, Moscow, Russia; b Molecular Pharmacology and Radiology Department, Russian National Research Medical University, Moscow, Russia

**Keywords:** Proliferative activity, migration, cell target, S100A4, dexamethasone

## Abstract

The study’s aim was to investigate the S100A4-mediated mechanisms of the regulation of tumor cell proliferation and migration in the human triple-positive breast carcinoma cell line MCF-7 (TPBC) and triple-negative breast carcinoma cell line MDA-MB-231 (TNBC). The proliferative activity of TNBC more than doubled during the incubation in the conditioned medium of TPBC. Extracellular S100A4 dose-dependently decreased the proliferative response of TPBC. TPBC negatively impacted the growth of TNBCs during their co-culturing. TPBC significantly decreased the migration activity of the TNBC cells while the S100A4 intracellular level in the TNBC was also decreasing. The decrease in the S100A4 intracellular level occurred due to the protein’s monomeric form while the contribution of the dimeric form into the overall S100A4 concentration in TNBC cells increased 1.5-2-fold. The S100A4 pathway in the intercellular communication between TNBC and TPBCs also included the dexamethasone-sensitive mechanisms of S100A4 intra- and extracellular pools regulation.

## Introduction

Breast cancer is now the most commonly diagnosed cancer in the world. The most recent global cancer burden figures estimate that there were 2.26 million incident breast cancer cases in 2020 and the disease is the leading cause of cancer mortality in women worldwide [1].

One of the intermediaries in the intercellular communication between heterogenous tumor cell lines is S100A4 protein. Calcium-binding S100A4 (Mts1) promotes the motility of tumor cells and, thus, plays an important role in tumor epithelial-mesenchymal transition, invasion, and metastasis. Both the expression of S100A4 and the invasion of MDA-MB-231 breast cancer cells were shown to be significantly higher as compared to those for MCF-7 breast cancer cells [2]. The expression of S100A4 is higher in TNBC with respect to non-TNBC patients [3]. Triple-negative breast cancer (TNBC) is an aggressive breast tumor subtype characterized by poor clinical outcome. TNBC is a complex heterogenous and aggressive cancer form, whose cells do not express hormonal receptors and HER2, and, thus, the tumors are resistant to hormonal therapy and to the therapy blocking HER2 receptors [4]. Patients with S100A4-positive tumors have inferior metastasis-free and overall survival compared to S100A4-negative. S100A4-expression was strongly associated with triple-negative subtype, metastasis-free and overall survival was numerically reduced in patients with S100A4-positive tumors [5]. However, sometimes TPBC shows more aggressive clinical features than TNBC. In a survival analysis over time, the TPBC showed a worse overall survival than TNBC 5 years posttreatment for breast cancer [6]. S100A4 is identified as significantly expressed in the biopsies of breast cancer hyperplasia and in malignant breast tumors. S100A4 is suggested to play a leading role in breast cancer invasion [2]. It was also suggested that S100A4 stimulates the microenvironment sustaining tumors [7]. At the same time, the level of S100A4 expression in metastatic cells depends on the tissue microenvironment. For instance, while comparing molecular profiles of the gene group associated with the metastasis of breast cancer cells in bones, it was demonstrated that the cells selected for their predominant metastasis in the bone tissue had the decreased S100A4 expression levels [8]. More detailed investigations established that S100a4 was the most inhibited gene in osteotropic breast cancer cells [9].

The mechanisms of reciprocal interaction between cell lines include inflammatory response mediators. S100A4-activated cells demonstrate the increase in the secretion of anti-inflammatory cytokines. In turn, secreted cytokines facilitate the formation of tumor-associated macrophages with protumorogenic functions [7]. The increased S100A4 expression in fibroblasts of amyotrophic lateral sclerosis patients suggests the contribution of S100A4-regulated pathways to neuroinflammation [10]. A recent study identified a number of changes in protein profiles of breast cancer cells co-cultured with human mature breast adipocytes. Hormone receptor-positive (MCF-7) and triple-negative (MDA-MB-231) breast cancer cells showed predominant down- and up-regulation of highly differentially regulated proteins, respectively, supporting the concept that reciprocal communication between breast cancer cells and cancer-associated adipocytes are heterogeneous and is likely to be breast cancer cell type-specific. The analysis showed that these regulated molecules participate in pathways related to metabolism, protein ubiquitination, and purine synthesis [11]. In some clinical practice cases during the tumor treatment, the triplet of positive breast cancer cells drastically changes and the immunohistology shows the disappearance of the cells with hormonal receptors. The tumor cells become more reactive and metastasis-prone. This process remains not well-understood. In the series of experiments on triple-positive MCF-7 breast cancer cells, it was demonstrated that the cells acquire a new phenotype under the influence of factors such as IL-1β and 5-AzaCdR. Specifically, the IL-1β influence induced the change of MCF-7 with typical epithelial morphology into elongated cells with structure similar to those of fibroblasts. IL-1β-stimulated cells started to express S100A4, to increasingly secrete active MMP-9 and MMP-2, and to invade the extracellular matrix. Under such circumstances, S100A4 expression allowed the cells to gain the ability to migrate [12]. The aim of this study was to investigate the S100A4-mediated mechanisms of auto- and paracrine regulation of: (i) proliferation; and (ii) tumor cell migration of both triple-negative MDA-MB-231 and triple-positive MCF-7 human breast cancer cells.

## Materials and methods

### Cell lines used in this study’s experiments

Human breast cancer cell lines: MCF-7 (American Type Culture Collection (ATCC)): Luminal A, estrogen receptor (ER)+, progesterone receptor (PR)+, HER2+; and MDA MB-231(ATCC): Basal, ER-/PR-/HER2- were cultured in Dulbecco’s Modified Eagle Medium (DMEM, Corning) supplemented with 10% fetal bovine serum (FBS, GE Healthcare-Hyclone), 100 units/ml penicillin, and 100 μg/ml streptomycin (Sigma Aldrich, USA), in a 37°C-incubator with 5% CO_2._

### Production of stimulated conditioned medium (S-medium) for the growth of activated breast cancer cells (MDA-MB-231 and MCF-7)

MCF-7 and MDA-MB-231 cells were mixed in the ratio of 100:1 and cultivated for 72 h until the formation of full cell confluency. The ratio and conditions were selected based on the preliminary experiments. The cells were maintained at 37°C and 5% CO_2_ in a humidified incubator. The medium then was extracted from the flask and filtrated through a 0.22-µm filter to remove any individual MCF-7 or MDA-MB-231 cells or cell debris. To produce stimulated conditioned medium (S-medium), the MDA-MB-231 and MCF-7 were cultivated together in the flasks retaining approximately the same 100:1 ratio until forming a stable cell culture. As the controls, we used the media from the flasks with individually cultivated MCF-7 (MCF-7-medium) and MDA-MB-231 (MDA-MB-231-medium) cells, cultivated under the same conditions.

### Assessment of cell proliferation and migration activity

The cell proliferation/viability was determined by CellTiter-Glo CellTiter 96® AQueous One Solution Cell Proliferation Assay (Promega Corporation) kit according to the manufacturer’s protocol. The relative cell viability was calculated as the percentage of untreated cells. Migration activity was determined by Transwell microchemotaxis chamber cell culture system (Costar). All assays were done in triplicate. In a control well, we added the equivalent volume (10 μl) PBS where the protein was dissolved. The upper and lower chamber compartments were separated by a tissue culture polycarbonate membrane (polyvinyl-pyrrolidone free; Nucleopore, Pleasanton, CA), 6.5 mm in diameter, with a pore size of 8 μm. MDA-MB-231 cells were resuspended in 200 μl of RPMI 1640 medium and carefully added to the upper compartment. 10% FBSs were placed in the total volume of 600 μl of RPMI 1640 medium in the lower compartment. After a 1-hour incubation period in a humidified incubator at 37°C and 5% CO_2_, the cell suspension in the lower compartment was harvested, and cell viability was determined using trypan blue. PBMCs were resuspended in 200 μl of RPMI 1640 medium and carefully added to the upper compartment. TNBC were transferred into the lower chamber of the Transwell system. Proteins S100A4-PGRPs complexes (10–7 M each) were transferred into the lower chamber of the Transwell system. Standard incubation for the formation of protein complexes was 30 min at 37°C in PBS (pH 7.4).

### Detection of S100A4 levels in conditioned media and cell extracts

The level of S100A4 secretion in the conditioned medium and this protein’s concentration in the cell extract were determined by the immunoferment method. After the cells were precipitated by centrifuging, the culture medium was collected in separate test tubes while the cell suspension was resuspended in Phosphate buffered saline (PBS) (рН 7.2–7.4) up to the cell concentration of 1 million per ml. The culture medium and the cells were centrifuged for 20 min at 2500 rpm, and then the S100A4 concentrations were measured in the supernatants using as a visualization control S100A4 recombinant protein and its rabbit antibodies manufactured by NeoMarkers (S100A4 Ab-8, Rabbit PAb RB-1804-A, Lot:1804A1505B NeoMarkers Fremont, CA), following other studies’ methods [13]. The expression of S100A4 was subjected to western blot analysis, and the polyclonal antibody to S100A4 were detected using the ChemiDoc MP Imaging System and Image Lab analyzer (BioRad, USA).

### Photodocumentation

The cell images were documented using a LEICA DFC 350 FX microscope’s light field with 400X magnification.

### Statistical analysis

Data are presented as a mean value ± SD of three independent experiments. A paired two-sample Student’s *t*-test was used for means to calculate statistical significance. P values equal or below 0.05 were considered as statistically significant.

## Results

### Reciprocal influence on proliferative activity and viability by TPBC and TNBC

**Figure 1.**
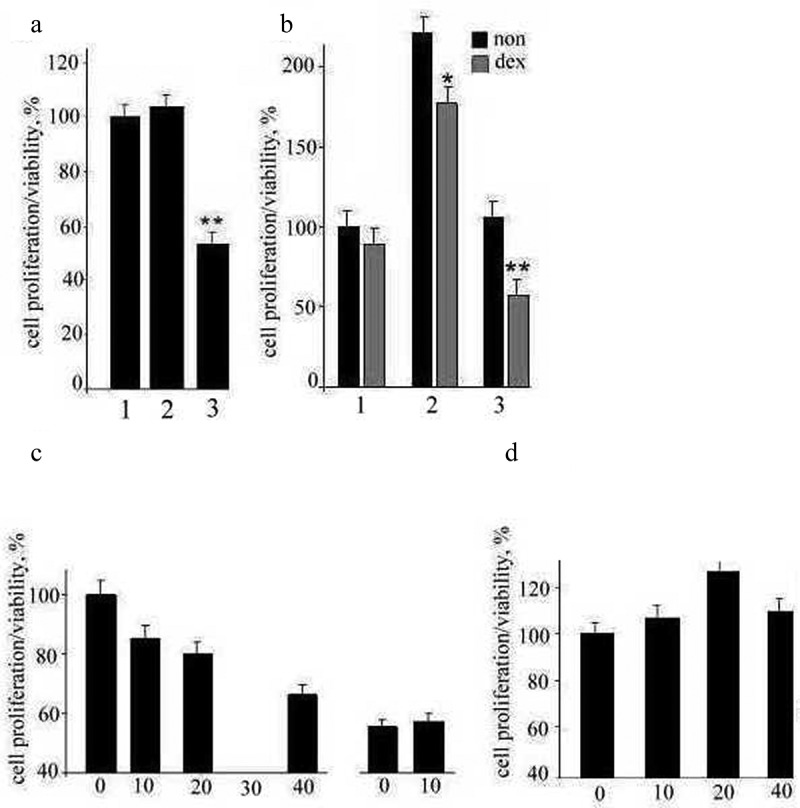
The proliferative activity of TPBC. The cells were incubated with: 1 – TPBC-medium; 2 – TNBC-medium; and 3 – S-medium (stimulated conditioned medium), *p < 0.05.B. The proliferative activity of TNBC without (black boxes) and under the presence (gray boxes) of dexamethasone (1 μmol/l): 1 – TNBC-medium; 2 – TPBC-medium; and 3 – S-medium, *p < 0.05.C. (Left panel, 4 boxes) The influence of S100A4 on the TNBC proliferative. activity: in TPBC-medium and under the 24-hour treatment by S100A4 in the concentration of 10, 20, and 40 ng per thousand cells;(Right panel, 2 boxes) The influence of S100A4 on the TNBC proliferative activity in TNBC-medium and under the 24-hour treatment by S100A4 in the concentration of 10 ng.D. The influence of S100A4 on TPBC growth. The proliferative activity of.TPBC was measured in TPBC-medium and under the 24-hour treatment by S100A4 in the concentration of 10, 20, and 40 ng per thousand cells.

To determine the influence of TNBC cells and their stimulated conditioned medium on the proliferation of TPBC, TNBC medium and stimulated conditioned medium (S-medium) were added to TPBC. As demonstrated in Figure 1(a), TNBC-medium did not produce statistically significant differences in MCF-7 cells proliferation (with a tendency for the increased proliferation). S-medium significantly (p < 0.05) decreased TPBC cells proliferation, approximately two-fold. These observations allowed us to propose that TNBC secrete factors influencing TPBC proliferation only when these cells are co-cultivated. To determine the influence of TPBC cells and their stimulated conditioned medium on the proliferation of TNBC, TPBC-medium and S-medium were added to TNBC. As demonstrated in Figure 1(b) the proliferative activity of TNBC increased more than two-fold when incubated in TPBC and did not change under S-medium. The addition of a synthetic analogue of glucocorticoid hormone, dexamethasone (1 μM), to the TPBC- and TPBC-medium led to the decrease in the cells’ growth, in average by 14–15%. The addition of dexamethasone to S-medium resulted in the significant decrease (almost two-fold) of the proliferative activity of TPBC cells. It can be observed that the co-cultivation of cells did not influence the proliferative activity of TPBC but made them susceptible to the steroid hormones’ effect.

### Influence of exogenous recombinant S100A4 on TNBC and TPBC growth

We proposed that S100A4 influences TNBC proliferation, given that TNBC cells express S100A4 protein and secrete that protein into the intercellular media. The addition of recombinant S100A4 protein into TPBC-medium in different concentrations resulted in the dose-dependent decrease of TNBC proliferation but did not influence that proliferation in TNBC-medium (Figure 1(c)). These findings confirmed the negative impact of S100A4 on TNBC proliferation. The treatment of TPBC with S100A4 protein led to a nonstatistically significant increase in these cells’ proliferation (Figure 1(d)).

### Influence of TPBC-medium and S-medium on the S100A4 expression and functional activity of TNBC

**Figure 2.**
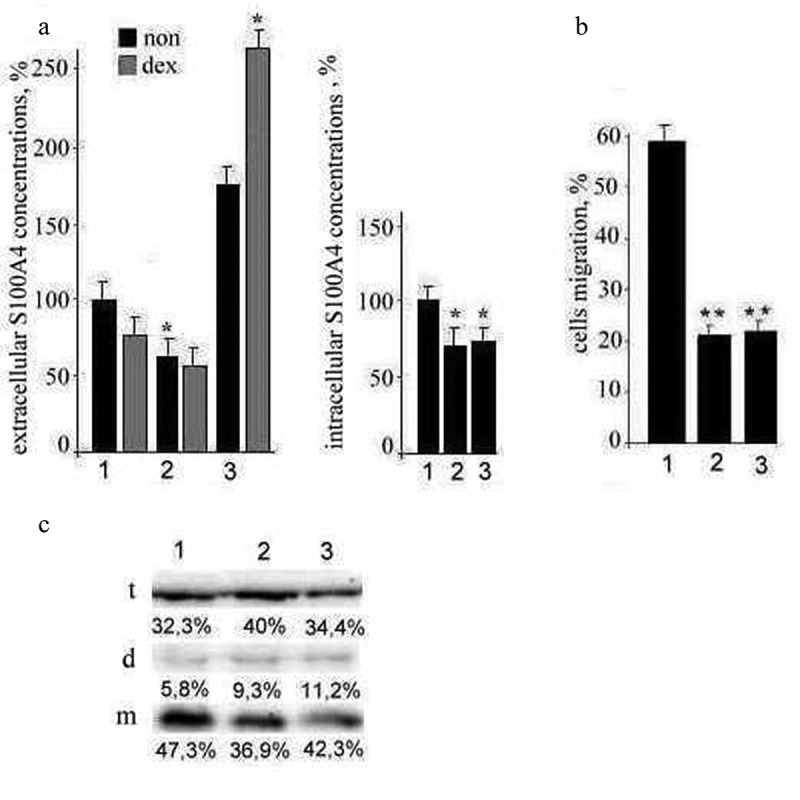
The measurement of intra- and extracellular S100A4 concentrations in TNBC and the migration activity of TNBC.

Further we investigated the influence TPBC on functional activity of TNBC. Additionally, we investigated the S100A4 expression by TNBC and the motility of the treated cells. The concentration of intracellular S100A4 in TNBC decreased under the incubation in both TPBC-medium and in S-medium. The concentration of extracellular S100A4 in TNBC decreased under the incubation in TPBC-medium and increased under the incubation in S-medium (Figure 2(а)). The treatment of the cells by dexamethasone and by S-medium increased (1.5-fold) the concentration of extracellular S100A4 (Figure 2 (а)). In turn, during these cells’ co-incubation, the concentration of intracellular S100A4 decreased due to the increased secretion of the protein. The migration activity of TNBC decreased 3-fold under the influence of the both media in comparison with TNBC-medium (Figure 2(b)). The biologic function of S100A4 is defined among others by the proteins’ ability to form oligomeric complexes. The western blot analysis demonstrated the increase of the monomeric S100A4 form in the cells in TNBC medium (24% increase) and in S-medium (15% increase) compared to TPBC medium. The monomeric S100A4 form percentages were: in TPBC – 36.9 (100%), TNBC – 47.3 (124%), and S – medium – 42.3 (115%). At the same time, the dimeric S100A4 form increased 1,5-fold in TPBC-medium and almost two-fold in S-medium. The tetrameric S100A4 form had the 24%-increase in TPBC medium and did not change in S-medium (Figure 2(с)).

## Discussion

We aimed to investigate the reciprocal influence of TPBC and TNBC cells. In our study, we compared the TNBC proliferative activity in the standard medium versus the medium where these cells were pre-co-cultivated with TPBC. We demonstrated that under normal circumstances TNBC secrete a factor that restricts their own growth.

One of the candidates for this factor can be S100A4 protein expressed by TNBC into the extracellular medium. The decrease in TNBC proliferation under the addition of S100A4 into their medium supports such hypothesis. The more than two-fold increase in TNBC proliferation in the medium containing individually cultivated TPBC suggests the existence of factors secreted by TPBC that bind extracellular S100A4. At the same time, the expression of S100A4 and the extracellular secretion of S100A4 in that medium were decreasing. However, in the medium where TPBC and TNBCs were pre-co-cultivated (stimulated medium), the secretion of S100A4 by TNBC increased and TNBC cells proliferation decreased (Table 1).

**Table 1.** Reciprocal relationships between phenotypically varying TNBC and TPBC and those relationships’ influence on proliferation and migration

fig	TPBC medium	TNBC medium	TPBC +TNBC medium
TPBC	Proliferation +	Proliferation +	Proliferation -
TNBC	Proliferation + Migration – exS100A4 -	Proliferation -	Proliferation - Migration – inS100A4 – exS100A4 +

S100A4: ex – extracellular; in – intracellular.

‘+’ – increase; “-“ – decrease; ‘±’ – no change.

In our series of experiments, the addition of dexamethasone into the stimulated medium led to the additional more-than-double increase in the secretion of S100A4 by TNBC, and the TNBC proliferation was significantly decreased. Our findings also demonstrate that the co-incubation made TNBC cells more sensitive to dexamethasone. The increased secretion of S100A4 protein was observed along with the decrease of intracellular S100A4, specifically of its monomeric form while its dimeric form increased 1.5-2-fold. The expression of tetrameric form, which has a regulatory function, did not change. These processes were associated with the decreased cells migration activity which might be explained by the ability of S100A4 to bind myosin IIA polypeptide chains forming complexes composed of one S100A4 dimer and a single myosin-IIA polypeptide chain [14]. As an alternative explanation, Neidhart and colleagues showed that monocytes treated with oligomeric S100A4 had increased levels of IL-1β, IL-6, and TNFα in response to lipopolysaccharide in comparison with intact cells [15].

The effects on TNBC cells proliferation and motility might not be direct but might be executed via stromal cells. For instance, the investigation of bilateral signal transmission and physical cell-to-cell interactions between fibroblasts and TNBC breast cancer cells demonstrated that cancer cells activate fibroblasts releasing an unknown factor. In turn, activated fibroblasts released one or several solvable signals that increased the speed of cancer cells aggregation and coalescence of the aggregates four-fold [16].

Other work demonstrated that S100A4 (Mts1) plays different roles in cancer progression depending on the different circumstances. S100A4 can have both prometastatic and antimetastatic functions [17]. In this study, TNBC inhibited their own growth while secreting S100A4 in the medium. Further investigations are required to understand the mechanisms behind the increased secretion of S100A4 by TNBC cells while they are co-incubated with TPBC.

S100A4 did not affect TPBC proliferation but slowed TNBC cells growth, the effect observed under small S100A4 quantities. It can be speculated that the increased secretion of S100A4 aims to compensate the factors secreted by TPBC and S100A4 inhibition or this increased secretion is a signal for other surrounding cells, for instance, fibroblasts. Our findings of the reciprocal influence of TPBC and TNBC cells can enhance our understanding of situations when an initial breast cancer is heterogenous and contains both triple-positive cells (that dominate and are actively stained during the immunohistology) and triple-negative cells (that are relatively small in numbers and can be easily missed). In these situations, under active treatment that targets triple positive cells, these cells’ presentation diminishes, while triple-negative cells start to actively proliferate. However, the antitumor treatment that will target simultaneously two different phenotypes (triple-negative and triple-positive) of tumor cells and will preclude their competitive survival, will also discourage the selection of triple-negative breast cancer cells.

Antitumor pharmacotherapy targeting the simultaneous inhibition of two different phenotypes (triple-negative and triple-positive) of tumor cells will allow avoiding the competitive advantage of one of these types and precluding the selection of triple-negative breast cancer cells.

## Data Availability

The data that support the findings of this study are available from the corresponding author, [EAD], upon reasonable request.
